# Operational parameters and their influence on particle-side mass transfer resistance in a packed bed bioreactor

**DOI:** 10.1186/s13568-015-0138-z

**Published:** 2015-08-14

**Authors:** Amir Hussain, Martin Kangwa, Nivedita Yumnam, Marcelo Fernandez-Lahore

**Affiliations:** Downstream Bioprocessing Laboratory, School of Engineering and Science, Jacobs University, Campus Ring 1, 28759 Bremen, Germany

**Keywords:** Packed bed reactor, Fluid side mass transfer, *Saccharomyces cerevisiae*, Alginate, Chitosan, Glucose

## Abstract

The influence of internal mass transfer on productivity as well as the performance of packed bed bioreactor was determined by varying a number of parameters; chitosan coating, flow rate, glucose concentration and particle size. *Saccharomyces cerevisiae* cells were immobilized in chitosan and non-chitosan coated alginate beads to demonstrate the effect on particle side mass transfer on substrate consumption time, lag phase and ethanol production. The results indicate that chitosan coating, beads size, glucose concentration and flow rate have a significant effect on lag phase duration. The duration of lag phase for different size of beads (0.8, 2 and 4 mm) decreases by increasing flow rate and by decreasing the size of beads. Moreover, longer lag phase were found at higher glucose medium concentration and also with chitosan coated beads. It was observed that by increasing flow rates; lag phase and glucose consumption time decreased. The reason is due to the reduction of external (fluid side) mass transfer as a result of increase in flow rate as glucose is easily transported to the surface of the beads. Varying the size of beads is an additional factor: as it reduces the internal (particle side) mass transfer by reducing the size of beads. The reason behind this is the distance for reactants to reach active site of catalyst (cells) and the thickness of fluid created layer around alginate beads is reduced. The optimum combination of parameters consisting of smaller beads size (0.8 mm), higher flow rate of 90 ml/min and glucose concentration of 10 g/l were found to be the maximum condition for ethanol production.

## Introduction

Ethanol is an alternative renewable, clean source of fuel currently used in combination with gasoline in the form of E10 and E20 (10 and 20% ethanol respectively) in transportation industries worldwide (Lei et al. 2011). Ethanol has a lower exhaust emission toxicity level when compared to that of petroleum (Matsushika et al. 2009). Currently, nearly 80% of industrial ethanol is produced via fermentation process (Shafaghat et al. 2011; Cha et al. 2014). Cell immobilization technology, is one useful technique that can be used to efficiently improve ethanol production by maintaining proper mass transfer and biological metabolic activity via the localization of intact cells to a defined region of space and preservation of catalytic activity for further biochemical process. Many methods like adsorption, covalent binding, cross linking, entrapment and encapsulation are widely used for immobilization (Terada et al. 2006; Borovikova et al. 2014; De Bari et al. 2013; Zhao and Delancey 2000), however, there has been an increasing interest in the research and development of advanced materials to obtain polymers with well-defined structures and specific chemical, physicochemical, mechanical and biological properties (Hussain et al. 2015). Natural and synthetic polymers such as cellulose, alginate, chitosan, agarose polyurethane, and polyacrylate are currently being used for cells (bacteria, yeast, fungi, and algae) immobilization for different bioprocesses (Gòdia et al. 1987; Duarte et al. 2013) and have potential application in bioethanol production due to their simplicity, cheap, non-toxic to cells and good mechanical properties. However, there are some drawbacks with their use, such as gel degradation, severe mass transfer limitations, low mechanical strength as it can cause the release of cells from the support and large pore size (Cascaval et al. 2012; Converti et al. 1985). Additionally, cell growth and gas production might rupture the carrier gel during fermentation (Rao et al. 1986). To overcome this, a combination of chitosan, a polycationic polymer and alginate, a polyanionic polymer is diffused into the alginate beads to provide a strong ionic interaction between chitosan amino groups and carboxyl groups of alginate which forms a polyelectrolyte complex (PEC) that gives more mechanical support to cells. It has also been shown that immobilization of yeast cells in alginate beads is the best strategies for improving industrial ethanol production and easy scale up of bioreactor (Chen et al. 2012; Chien and Sofer 1985), as in bioreactors substrates are effectively bio-converted to the desired products under the microbial cells or enzyme activity (Hussain et al. 2015).

The main problem in bioreactor system is the inadequate substrate, product and other metabolites transfer towards cells and out of the immobilizing matrix (Nunez and Lema 1987). Recently scientists have shown great interest on the application of packed bed bioreactors for production of bio-ethanol due to its low manufacturing and operating cost (Galaction et al. 2012; Rivaldi et al. 2008).

**Fig. 1:**
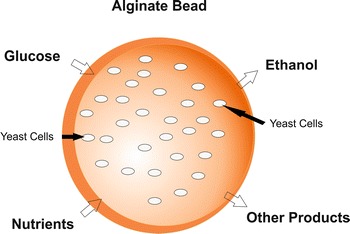
Schematic presentation of mass transfer of substances in and out of alginate bead.

A comprehensive understanding of mass transfer in immobilized system using packed bed bioreactor is required to achieve maximum productivity as well as the performance of bioreactor (Warnock et al. 2005; de Jong et al. 2012). There are two major steps involved in substrate transfer (Fig. 1) in immobilized alginate beads; (1) Transfer of substrate from homogeneous bulk liquid to external surface of beads passing through hypothetically stagnant liquid film around the beads, also known as external mass transfer. (2) Transfer of substrate to microorganisms through the pore, also known as internal mass transfer.

In immobilized cell system, substrates are consumed and metabolites are produced that leads to the generation of a concentration gradient. Interphase concentration gradients are commonly present in liquid–solid system and can be decreased by reducing beads size and increasing the flow rate of fluid (Perego and Peratello 1999).

In our study on packed bed bioreactor with immobilized *S. cerevisiae* cells in alginate shows that it is possible to have efficient external mass transfer without loss of cell growth and physiology by selecting optimum flow rate. It is possible to continue investigating operational performance of the immobilized packed-bed bioreactor in the course of physiological and biochemical studies on the substrate uptake of immobilized yeast cells (Hussain et al. 2015). In this study the reactor was operated in batch mode fermentation; yeast physiology and internal mass transfer behavior in packed bed reactor were monitored in close relation to parameters such as bead size, medium flow rate, substrate concentration and different support materials like alginate beads with and without chitosan coating.

## Materials and methods

### Microorganism

*Saccharomyces cerevisiae* (baker yeast) yeast strain was purchased from DHW Vital Gold, Nürnberg, Germany and the samples were kept at 4°C.

### Fermentation medium and cultivation

For this study, minimal media was utilized in the cultivation process, prepared with 6.7 g/l yeast extract nitrogen base without amino Acid, 1.7 g/l ammonium acetate and glucose (4 and 10 g/l) were prepared separately and mixed after sterilizing (121°C, 20 min.). The following different amino acids were mixed to prepare “Amino Acid mixture” (100X); 200 mg L arginine, 1,000 mg l-aspartic acid, 1,000 mg l-Glutamic acid, 300 mg l-lysine, 500 mg L phenylalanine, 4,000 mg l-serine, 2,000 mg l-threonine, 300 mg l-tyrosine, 1,500 mg L valine. All components were dissolved in distilled water by adjusting pH 10 with 0.1 N NaOH and used 0.2 μm filter for sterilization and 10 ml of amino acids solution was added to a final 1 L media.

### Calcium alginate beads preparation and yeast immobilization

During preparation of calcium alginate beads a sterile sodium alginate solution 2.5% (w/v), autoclaved at 121°C, for 15 min, was prepared in 50 mM phosphate buffer at pH 7. The cell suspension (3%) was mixed with alginate solution for immobilization of baker yeast. In case of beads preparation, alginate-yeast solution was drop by drop allowed to dip using 1 ml pipette tip into 200 ml, 180 mM CaCl2. Beads were let to harden in this solution for 1 h. Further, beads were rinsed three times with sterile 2% NaCl solution and then with sterile water. The alginate beads with diameters 0.8, 2 and 4 mm were used in experiments. For the preparation of chitosan coated alginate beads, the above prepared beads were dipped in sterilized chitosan solution (3% chitosan, 0.1 N HCl, pH 5) for 10 min and later washed 3 times with sterile water.

### Packed bed reactor and beads packaging

A packed bed bioreactor (100 ml) was used for experiments and purchased from Medorex GmbH, Noerden-Hardenberg, Germany. The bioreactor column has a 2 cm diameter glass vessel for beads package, with one end close and other closed by rubber plug. The reactor was 2/3 filled with beads and temperature was kept at 35°C using a water bath. The immobilized yeast was grown on minimal media with varying factors: glucose (4 and 10 g/l), flow rate (4, 30 and 90 ml/min.) and alginate bead with and without chitosan coating while factors like initial cells amount (3%) and temperature (35°C) were kept constant.

### Glucose consumption measurements

The DNS method was used for measurements of immobilized yeast glucose consumption. For each measurement, 0.5 ml sample and 0.5 ml DNS solution were mixed in a 1.5 ml Eppendorf tube, vortex for 10 s, and incubated for 10 min at 90°C. After incubation, 40% 0.16 ml potassium sodium tartrate was added, mixed by vortex and placed on ice for 3 min. Two hundred microliter of each sample was measured at 575 nm. The obtained results were compared with calibration curve of different glucose concentration to get actual concentration.

### Ethanol production measurements

The concentration of ethanol produced in fermentation broth as well as calibration curve was measured with the same method as in previous paper (Hussain et al. 2015). The fermentation broth samples (each having volume 600 μl) were collected, transferred to an Eppendorf tube and centrifuged at 9,000 rpm for 5 min to pellet the cells. Later, 500 μl of the clear supernatant were transferred into a new tube without disturbing the cell pellet, and 5 μl of 1% *n*-butanol was added as an internal standard. After vortexing the samples for 30 s, 1 ml of 25% ethyl acetate was added with a further 5 min vortex. The samples were centrifuged for phase separation, at 5,000 rpm and the organic phase was used for gas chromatography (GC). The gas chromatograph equipped with flame ionization detector (FID) was used for sample measurements. The columns used were the 30 mm and 0.25 mm CP WAX—57CB (Santa Clara, CA, USA). The column temperature was initially maintained at 120°C for 2 min and later the oven temperature was increased at a rate of 10°C/min until it reached 150°C. The temperature of injector and detector were kept at 150°C and 200°C, respectively. The flow rate for carrier gas (Helium) was set at 30 ml/min. The injection sample volume was 2 μl. Each experiment was repeated thrice and the reported value was the mean average.

## Results

### Effect of glucose concentration, flow rate and beads size on lag phase

**Fig. 2:**
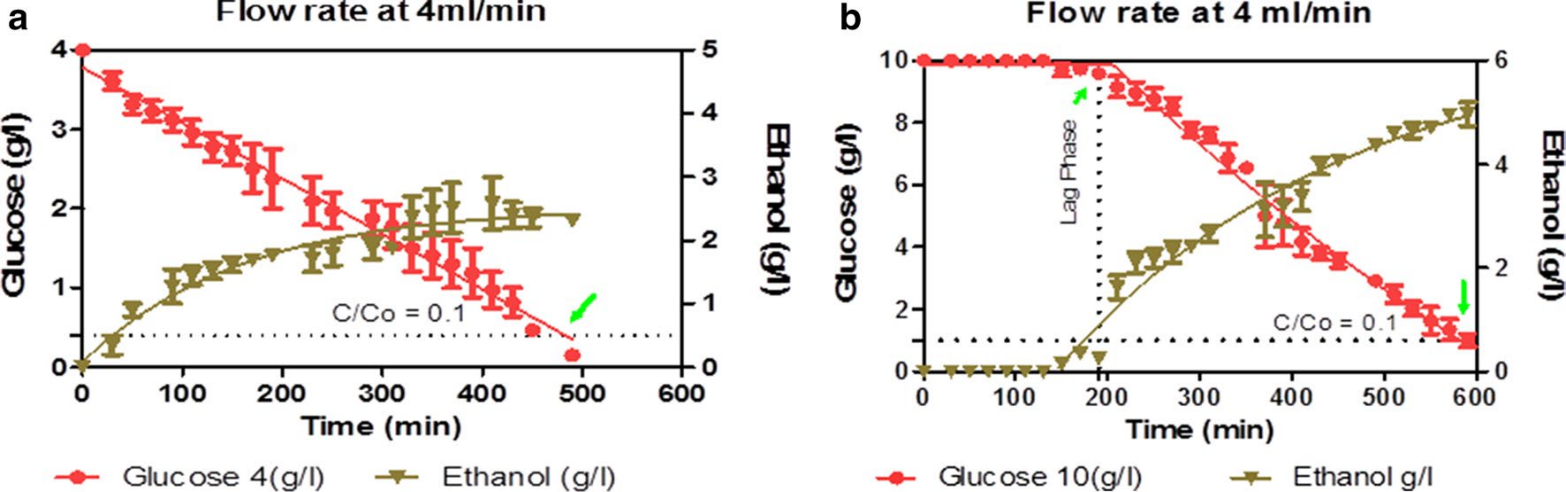
Effect of glucose concentration on lag phase. No lag phase was observed when using glucose concentration of 4 g/l.

**Fig. 3:**
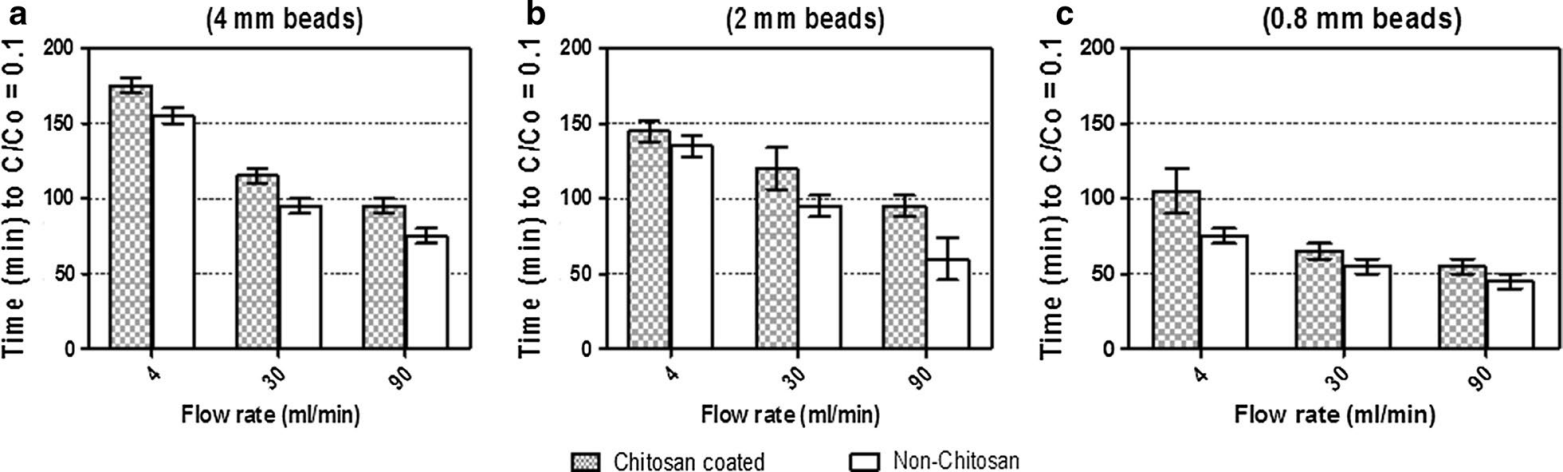
Effect of flow rate, glucose concentration and beads size on lag phase.

Figure 2a, b show the results obtained on glucose consumption 4 and 10 g/l, respectively, and ethanol production with time. The results shows that when using glucose concentration of 4 g/l, no lag phase was observed (Fig. 2a) while with 10 g/l the lag phase lasted for about 190 min (Fig. 2b). Figure 3 represents the bar chart results obtained on three different parameters; flow rate (4, 30 and 90 ml/min), bead size (0.8, 2 and 4 mm) and on glucose concentration of 10 g/l. These parameters have a significant effect on internal mass transfer and can be observed in the form of lag phase at the start of fermentation process (Fig. 2). Since there was no lag phase when using glucose concentration of 4 g/l, the bar chart results were omitted. Figure 3, shows that the duration of lag phase on bead types decreases by increasing flow rate and decreasing the size of beads, moreover, longer lag phase was found at higher glucose concentration (Figs. 2, 3). The maximum time on lag phase was found to be 190 min at lower flow rate of 4 ml/min and 90 min at higher flow rate 90 ml/min when using 10 g/l of glucose in medium with 4 mm size of beads (Figs. 2, 3). By decreasing the size of beads to 2 and 0.8 mm, duration of lag phase decreased as while. Lowest time was 50 min when using 0.8 mm beads at 10 g/l glucose medium (Fig. 3). It was observed that by decreasing glucose concentration from 10 to 4 g/l, lag phase tends to decrease (Fig. 2). No lag phase was found at glucose concentration of 4 g/l (Fig. 2) with non-chitosan coated beads have shorter lag phase as compared to coated beads, indicating an improved internal mass transfer effect and less inhibition by glucose.

### Effect of flow rate, glucose concentration and beads size on glucose consumption

**Fig. 4:**
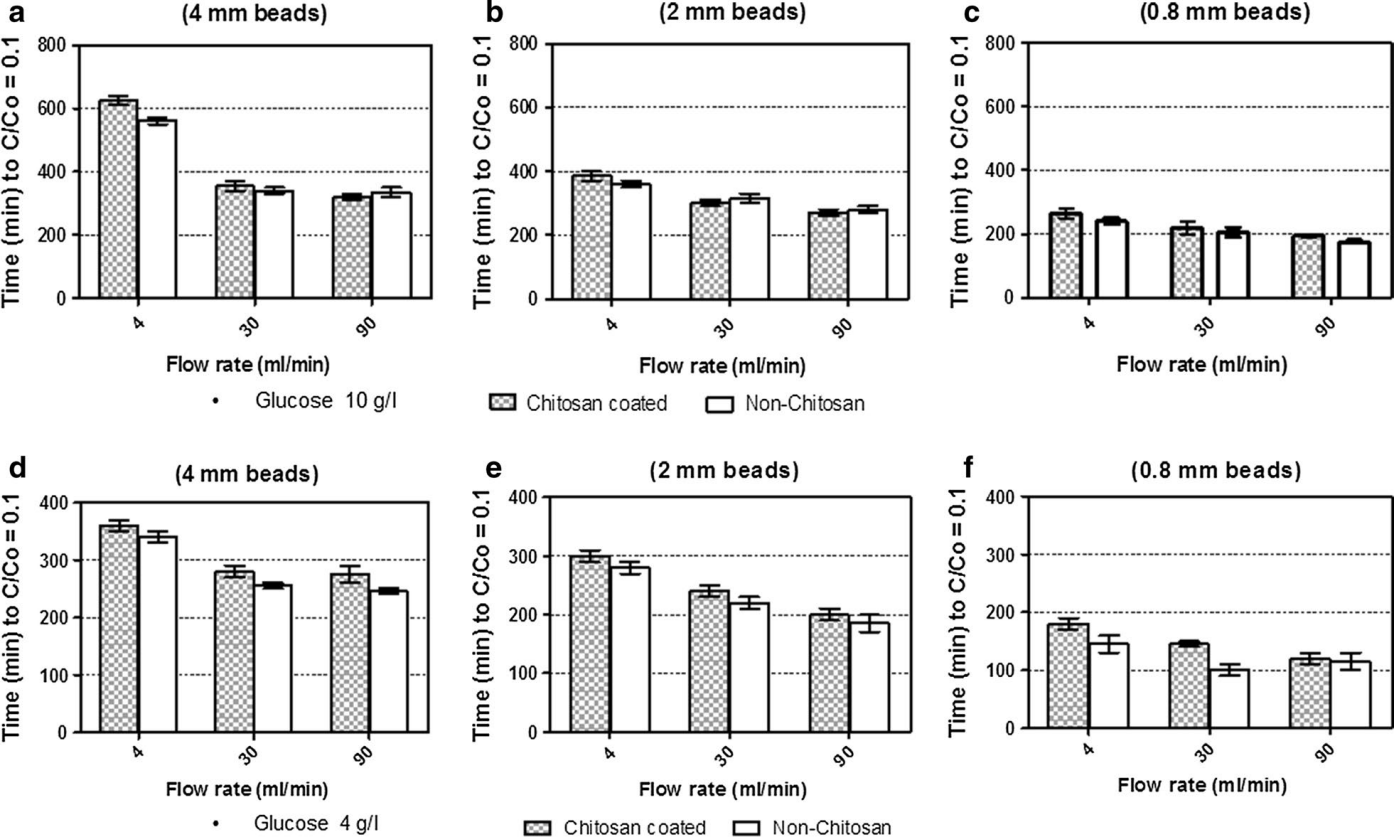
Effect of flow rate, glucose concentration and beads size on glucose consumption. **a**–**c** and **d**–**f** represent data obtained from using glucose concentration of 10 and 4 g/l, respectively.

To understand the internal mass transfer properties of chitosan and non-chitosan coated beads, glucose consumption up to the level of C/Co = 0.1 was measured. Where Co represents the initial glucose concentration at time zero, C is the concentration at a particular time and 0.1 (10%) is the remaining glucose in the media. By varying the flow rate 4, 30 and 90 ml/min (Fig. 4, time for glucose consumption fluctuate. The major difference in glucose consumption behavior was observed when using both types of beads at higher flow rate like 90 ml/min. The time for glucose consumption of beads 4 and 2 mm at flow rate 30 and 90 ml/min was rather equal using 4 and 10 g/l (Fig. 4). With beads having no layer of chitosan, glucose consumption time tends to decrease by increasing flow rate. Figure 4 clearly shows that glucose consumption time sharply reduced by decreasing the size of beads. For beads size 4, 2 and 0.8 mm, at glucose concentration 4 and 10 g/l at lower flow rate (4 ml/min), consumption time ranges from 350 to 600 min, 300 to 400 min and 150 to 260 min respectively. By increasing flow rate (30 ml/min), it ranged from 250 to 350 min, 220 to 320 min and 100 to 220 min with respect to size of beads and glucose concentration, whereas higher flow rate 90 ml/min further reduces consumption time by approximately 100 min for each bead size (Fig. 4).

### Effect of flow rate, glucose concentration and beads size on ethanol productivity and yield

The yeast cells inside the beads were maintained to be uniform by using minimal medium where growth was at its minimal. Using three types of beads (0.8, 2 and 4 mm) experiments were conducted having initial glucose concentrations 4 and 10 g/l and flow rate 4 and 90 ml/min with dilution rate of 0.2 and 4.5 h^−1^ respectively. The two important factors like flow rate and dilution rate effect at different glucose concentration on ethanol productivity as well as on ethanol yield is presented in Table 1. An optimal ethanol productivity of 32.4 g/(g h) was obtained when using 2 mm beads at D of 4.5 h^−1^ at glucose concentration of 10 g/l. By using initial glucose concentration 4 and 10 g/l, ethanol productivity increases linearly with the dilution rate from 0.2 to 4.5 h^−1^.

**Table 1:** Ethanol productivity and yield by yeast strains

Flow rate (ml.min^−1^)	Dilution rate (−)	Glucose conc. (g/l)	Ethanol productivity (g/l h)			Ethanol yield (g g^−1^)		
			0.8 mm	2 mm	4 mm	0.8 mm	2 mm	4 mm
4	0.2	4	0.56	0.50	0.40	0.93	0.83	0.80
4	0.2	10	1.18	1.00	0.56	0.73	0.62	0.60
90	4.5	4	15.75	13.90	10.8	1.16	1.03	0.63
90	4.5	10	32.40	27.00	17.10	0.90	0.75	0.48

Beads size 0.8, 2 and 4 mm.

Ethanol productivity = D × P (at: 300 min).

Ethanol yield Y(p/s) = Pl − P_o_/S_o_ − Sl (at: 300 min).

*D* dilution rate, *P* product concentration

## Discussion

In homogeneous catalytic reaction, mass transfer effect is considered as negligible because reactant (glucose) and catalysts are in one phase while in heterogeneous, both are in different phases. The catalyst normally is in solid phase and reactants are in liquid phase and the reaction is dependent on the mass transfer (Klaewkla et al. 2011), meaning that reaction only takes place when reactants are transferred to the catalytic reaction site by diffusing across external fluid layer around the catalyst (external mass transfer) into pores within catalyst (Internal mass transfer). In heterogeneous reaction, improvement of mass transfer and elimination of limitations are desired objectives (Salmon and Robertson 1987). In this research, our purpose was to understand the mechanism of internal mass transfer effect on immobilized system used for ethanol production and the performance of packed bed bioreactor. Internal mass transfer resistance is strongly depended on these parameters: glucose concentration in the medium, coating on alginate beads (chitosan and non-chitosan coated beads), flow rate and size of beads. However, it can also be compensated to a certain extent, by varying the above mentioned parameters and its effect can be observed on lag phase at the start of fermentation, consumption of glucose in medium and production of ethanol.

We discussed lag phase in previous paper (Hussain et al. 2015), as adaptation time of yeast within new environment at the start of fermentation. The reason observed might be the occurrence of significant mass transfer effects when reaction takes place inside the catalyst (beads) (Salmon and Robertson 1987; Anselme and Tedder 1987). The effect of internal mass transfer resistance on lag phase could be understood from Figs. 2 and 3 which shows that lag phase duration for both types of beads (chitosan and non-chitosan coated) sharply reduced by reducing the size of beads. In literature, there is enough data on the inhibition of yeast growth and metabolic activities by high initial substrate concentration (Galaction et al. 2010; Lee et al. 2012), and the result depicts that substrate or product inhibition phenomenon can limit the efficiency of ethanol production and leads to enhance the lag phase of fermentation process. (Talebnia and Taherzadeh 2007) also observed the limitation of transfer of substrate into the centre of beads and toxic metabolite out of it.

Varying the size of beads is an additional factor which may control internal mass transfer. By reducing this factor, the distance for reactants to reach active site of catalyst and the thickness of fluid created layer around alginate beads is reduced. On the other hand, when beads are in large size, substrate is depleted in the center and core of beads deprived of substrate (Salmon and Robertson 1987). Moreover, internal mass-transfer limitations can be overcome with high liquid flow rate that reduces the fluid layer around the beads and increasing the surface concentration, which in return effectively increases its diffusivity. All these parameters are most effective to reduce internal mass transfer limitations as we have observed in Figs. 2, 3. The flow rate of 90 ml/min is more effective than 4 ml/min in reducing the lag phase time to 100, 50 and 40 min for 4, 2 and 0.8 mm size of beads using 10 g/l of glucose in medium. The hydrodynamics of the medium exhibits an important influence on glucose conversion and transfer processes (Galaction et al. 2012).

It was also observed that lag phase is dependent of glucose concentration and tends to decrease by reducing from 10 to 4 g/l in the medium. Even at 4 g/l glucose concentration, no lag phase was observed indicating less internal mass transfer or concentration gradient effect by the substrate. The dependence of lag phase on glucose concentration (Fig. 2) might be the result of substrate diffusion and the concentration gradient between surface and inner regions of beads (Hussain et al. 2015; Zhao and Delancey 2000).

Data analysis of the experiments show that time for glucose consumption at flow rate 30 and 90 ml/min was rather equal when using 4 mm size of beads and it tends to decrease by decreasing the size of beads from 2 to 0.8 mm, this might be due to the decrease in the concentration gradient in and outside the beads (Galaction et al. 2011). The concentration gradient is the difference of (substrate or product) concentration between two phases. External concentration gradient is the difference of concentration between the bulk liquid and external surface of the beads and it can never be observed without larger internal gradients within the beads (Salmon and Robertson 1987) where the product inside the beads diffuses out under the influence of concentration gradient.

Not so much significant difference of glucose consumption time was noticed between chitosan-coated and non-coated alginate beads at higher flow rate (30 and 90 ml/min) when compared to lower flow rate (4 ml/min) especially when using smaller bead size (0.8 mm). This might be due to the removal of diffusional limitations in and around the beads. In literature, it was found that substrate conversions yield is reduced by increasing the size of biocatalyst particles and glucose initial concentration due to higher resistance of the substrate internal diffusion and it was concluded that magnitude of resistance to the internal diffusion is directly related to the particle size and glucose concentration gradient (Galaction et al. 2010; Engasser and Horvath 1973).

It has been observed that higher ethanol productivity could be achieved by increasing flow rate and glucose concentration because of improved mass transfer properties or reduction of internal substrate diffusional resistance by varying the size of beads and higher flow rate (Chien and Sofer 1985; Zhao and Delancey 2000). No significant difference was observed in ethanol productivity for both types of beads at lower flow rate i.e. 4 ml/min and was higher at higher flow rate (90 ml/min). By increasing liquid velocity ethanol productivity was observed at its maximum because it enhances the surface substrate transfer and reduce the substrate gradient effect inside the beads (Bangrak et al. 2011).

Higher glucose concentration has a major role to achieve maximum ethanol productivity (Converti et al. 1985). Internal mass transfer resistance has been observed at lower flow rate that induces glucose accumulation as substrate inhibitory effect and due to low diffusion rate of ethanol from the inner region of beads to the medium, the product inhibition also might be generated (Rotaru et al. 2011; Engasser and Horvath 1973; Prasad and Mishra^1995^).

Furthermore, in Table 1, beads with 0.8 mm size have higher yield than 2 mm type at all flow rate and glucose concentration due to reduction of intra-phase mass transfer limitations. Reduction in size of beads until no longer intra-phase mass transfer limitations exists, enhances the ethanol productivity which was also observed by (Duarte et al. 2013; Pilkington et al. 1998; Boersma et al. 1979). Furthermore in both types of beads, the addition of glucose has also been observed to have an effect on the ethanol productivity that might be due to increased substrate diffusional resistance and development of concentration gradient. Another reason might be the inhibition or reverse of reaction because of higher rate of reaction upon increasing substrate concentration and also observed (Nikolić et al. 2009) a significant decrease in ethanol yield on addition of sugar concentration in fermentation medium. Furthermore, it was also found that intra-phase resistance is directly related to the glucose concentration gradient which induces substrate inhibition (Galaction et al. 2010).
